# Higher frequency of social learning in China than in the West shows cultural variation in the dynamics of cultural evolution

**DOI:** 10.1098/rspb.2014.2209

**Published:** 2015-01-07

**Authors:** Alex Mesoudi, Lei Chang, Keelin Murray, Hui Jing Lu

**Affiliations:** 1 Department of Anthropology and Centre for the Coevolution of Biology and Culture, Durham University, South Road, Durham DH1 3LE, UK; 2 Department of Educational Psychology, Chinese University of Hong Kong, Ho Tim Building, Shatin, New Territories, Hong Kong; 3 School of Biology and Centre for Social Learning and Cognitive Evolution, University of St Andrews, Harold Mitchell Building, St Andrews, Fife KY16 9TH, UK; 4 Department of Applied Social Sciences, Hong Kong Polytechnic University, Hung Hom, Kowloon, Hong Kong

**Keywords:** asocial learning, cultural evolution, cultural transmission, innovation, social learning

## Abstract

Cultural evolutionary models have identified a range of conditions under which social learning (copying others) is predicted to be adaptive relative to asocial learning (learning on one's own), particularly in humans where socially learned information can accumulate over successive generations. However, cultural evolution and behavioural economics experiments have consistently shown apparently maladaptive under-utilization of social information in Western populations. Here we provide experimental evidence of cultural variation in people's use of social learning, potentially explaining this mismatch. People in mainland China showed significantly more social learning than British people in an artefact-design task designed to assess the adaptiveness of social information use. People in Hong Kong, and Chinese immigrants in the UK, resembled British people in their social information use, suggesting a recent shift in these groups from social to asocial learning due to exposure to Western culture. Finally, Chinese mainland participants responded less than other participants to increased environmental change within the task. Our results suggest that learning strategies in humans are culturally variable and not genetically fixed, necessitating the study of the ‘social learning of social learning strategies' whereby the dynamics of cultural evolution are responsive to social processes, such as migration, education and globalization.

## 1. Introduction

When is it adaptive to copy others, rather than go it alone? While social learning and social influence have been topics of longstanding interest in the social sciences [1,2], only recently have evolutionary anthropologists, biologists and psychologists examined the adaptive basis of social learning (copying solutions to problems from others) relative to asocial learning (solving problems independently, e.g. via trial-and-error), using both formal theoretical models and controlled laboratory experiments in multiple species [3]. While initially social learning was seen as informationally ‘parasitic’ [4], with social learners ‘scrounging’ information produced at some cost by asocial learners, recent models have revealed a range of conditions under which social learning can theoretically enhance the fitness of both individuals and populations [5–10]. Moreover, humans are thought to possess social learning of uniquely high fidelity, allowing us to accumulate socially learned knowledge and skills over successive generations in a way other species cannot [11–14]. This cumulative cultural evolution, it is argued, has allowed our species to adapt rapidly to novel and diverse environments across the planet [13,14].

However, when the predictions of theoretical models have been tested using controlled laboratory experiments with real people, several independent research groups have found that people copy less than they should do if they were maximizing their payoffs [15–22]. This has been found with participants from the UK [15,18], USA [17,20,21], Germany [16] and Sweden [19] using different tasks, as well as in various games conducted by experimental economists in Western Europe and USA [22], suggesting that this finding is not a peculiarity of a particular task or procedure.

It may, however, be a peculiarity of the participant sample used in these studies, who are all from so-called WEIRD (Western, Educated, Industrialised, Rich, Democratic) countries [23]. Indeed, several lines of circumstantial evidence suggest that human social learning is cross-culturally variable, with people in the West less likely to copy others than people from East Asia [24]. Western education emphasizes individual discovery and creativity, whereas East Asian education emphasizes rote learning from authority [25]. The adoption of consumer products shows less social influence in Western than East Asian countries [26]. Westerners are described as more individualistic/independent, while East Asians are described as more collectivistic/interdependent [27], dimensions which intuitively map on to asocial and social learning, respectively. Finally, experiments conducted by social psychologists have shown greater social influence in collectivistic East Asian societies than individualistic Western societies [28], although the tasks used in such studies are limited in their ability to determine the *adaptiveness* of different learning strategies due to participant-deception and simple tasks with solutions that are intuitively obvious [21].

The possibility that human learning strategies are cross-culturally variable not only potentially resolves the aforementioned mismatch between theory and data, but also challenges the explicit or implicit assumptions of many theoretical models that learning strategies are species-universal, are under fixed genetic control, and change via natural selection. It is often assumed, for example, that ‘an individual's position on this continuum [of social vs. asocial learning] is a genetically heritable trait’ [5, p. 131], or that ‘Which [learning] strategy is used is genetically determined for each individual’ [6, p. 728]. Similarly, claims by comparative researchers that ‘humans' are unique in their social learning capacities compared to other species [11,12] implicitly extrapolate from one cultural sample to the entire human species. To date, few models have explicitly examined the social learning of learning strategies [29,30], and few experiments have examined the adaptiveness of social learning in non-WEIRD populations [10,31]. Of the latter, one study [10] found that Japanese participants successfully avoided maladaptive producer–scrounger dynamics through their use of social learning, although the study did not test whether the level of social learning was optimal. Another study in Bolivia [31] found similar sub-optimal social information use as in Western populations. In both studies, the lack of Western control groups precludes a direct comparison between cultures.

**Figure 1.**
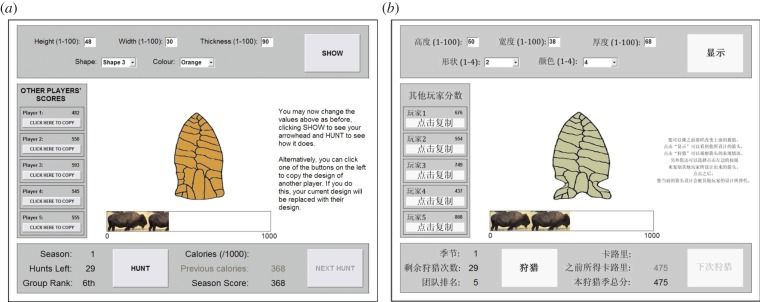
Screenshots of the virtual arrowhead task in (*a*) English and (*b*) Chinese. Participants engage in trial-and-error asocial learning by directly manipulating the attributes (height, width, thickness, shape and colour) using the boxes along the top of the screen, or copy one of the asocial-learning-only demonstrators using the buttons on the left of the screen. Once the participant is happy with their design, they click the HUNT button to test their arrowhead and receive a score out of 1000 calories. This is added to their cumulative season score, and they are also given their group rank relative to the five demonstrators based on season scores. (Online version in colour.)

Here we provide the first direct cross-cultural East–West comparison of the adaptiveness of human social learning, with no participant-deception and with a challenging task with no intuitively obvious solution. This task is designed to reflect real-life learning about complex, cognitively opaque technological artefacts typical of cumulative culture, and has previously been shown to elicit lower-than-optimal levels of social learning in a Western sample [15]. It was administered in four cultural groups along a continuum of Western–Eastern influence: (i) White British students from the United Kingdom (group ‘UK’); (ii) Chinese immigrants raised in China currently studying in the UK (‘CI’); (iii) Chinese students raised and studying in Hong Kong (‘HK’), and (iv) Chinese students raised and studying in the culturally traditional and homogeneous Chao Zhou region of the Chinese mainland (‘CM’). Via a computer program, participants designed ‘virtual arrowheads' over three seasons each comprising 30 hunts, or opportunities to improve and test their arrowhead (figure 1). After each hunt, they received a payoff in calories and were rewarded monetarily based on their accumulated payoff over all three seasons. On each hunt, participants could copy the design of one of five asocial-learning-only demonstrators, given information about those demonstrators' performance. This permitted payoff-biased social learning [15] and eliminated potentially maladaptive producer–scrounger dynamics [9]. Seasons 1 and 2 featured constant environments (the optimal arrowhead values did not change), while Season 3 introduced within-season environmental change which theory predicts should reduce reliance on social learning [32], and which was used to test the within-task flexibility of learning strategies. Our key questions are whether all four cultural groups exhibit the same or different frequencies of social learning, and how this impacted upon their payoffs.

## 2. Material and methods

### (a) Participants

Seventy-six British participants (40 female, mean age 20.38 years, s.d. = 2.71) and 70 recent Chinese immigrants to the UK (48 female, mean age 20.49 years, s.d. = 2.33) were recruited from Durham University's student population. The Chinese immigrants were almost exclusively from China's three largest cities, Shanghai, Beijing and Guangzhou, and moved to the UK within the previous 1–2 years to attend university. The Hong Kong sample comprised 73 participants (34 females, mean age 20.26, s.d. = 1.77) studying at the Hong Kong Polytechnic University. The Chinese mainland sample comprised 73 participants (37 females, mean age 21.15, s.d. = 1.37) studying at Chao Zhou Normal University in Chao Zhou, a relatively small city of 2.6 million inhabitants in the same province (Guangdong) as Hong Kong and who spoke the same language (Cantonese) as the Hong Kong sample (see the electronic supplementary material for further details of sample comparability). Five additional asocial-learning-only demonstrators were recruited for each of the four cultures.

Participants were paid a flat fee for turning up, with monetary increments added according to how well they performed. UK and CI participants were paid £8 for taking part, with up to £4.20 more available according to their success in the task (7p for every 1000 calories obtained after the first 10 000 calories in each season). HK participants were paid a flat fee of HK$100 (£8) and increments of HK$5 up to HK$50 (£4). CM participants were paid RMB60 (£6) and RMB3 increments up to RMB30 (£3).

### (b) Task/procedure

All participants completed a computer-based task to design a virtual arrowhead which is then used on a series of hunting trips (see [15], figure 1 and the electronic supplementary material for screenshots of task and instructions). Participants enter five attributes that independently determine their arrowhead design: three continuous (height, width and thickness) ranging from 1 to 100 arbitrary units, and two discrete (shape and colour) which can each take one of four values. The overall effectiveness of the arrowhead is a function of how close its attributes are to hidden optimal values (except colour, which was neutral). These optimal designs can be seen as those most suited to the participant's particular ‘environment’. The continuous attributes each had bimodal fitness functions creating a multimodal fitness landscape, such that there were eight locally optimal arrowhead designs of varying maximum fitness. The global optimum gave a score of 1000 calories. Seven other peaks gave slightly lower maximum scores. The greater the deviation from these optima the lower the score. Small normally distributed random error was added to the scores to increase realism.

The aim of the task for the participant is to accumulate as high a score as possible over a series of trials (‘hunts') by locating the optimal value of each attribute. Following a five-hunt asocial-learning-only practice session, there were three seasons of hunting, each comprising 30 hunts, or 30 opportunities to modify and test the arrowhead. Participants improve their design either by trial-and-error asocial learning, i.e. modifying arrowhead attributes in response to changes in score over successive hunts, or social learning, i.e. copying the design of another participant. Following [15], we ran separate groups of asocial-learning-only demonstrators that experimental participants could subsequently copy, rather than allowing participants to copy each other in real time. This design provided more comparable data across participants and eliminated producer–scrounger dynamics by ensuring the constant presence of pure information producers. To avoid ingroup–outgroup effects and increase external validity we ran separate groups of five demonstrators for each of the four cultures, such that UK participants copied UK demonstrators, HK participants copied HK demonstrators, etc. Participants could choose, on each hunt except the first of each season, to copy the arrowhead design that a demonstrator had used on the equivalent hunt (e.g. on hunt 5, participants could copy the arrowhead that one demonstrator had used on their hunt 5). Participants were informed of the cumulative score of each demonstrator on the equivalent hunt, allowing (but not requiring) participants to preferentially copy the highest-scoring demonstrator. Choosing to copy entailed the replacement of the participant's arrowhead with that of the demonstrator with no opportunity to further modify the arrowhead on that hunt, to prevent both social and asocial learning occuring on the same hunt. Demonstrators and all experimental participants experienced identical season/hunt structures and fitness functions.

After participants have chosen whether to modify their arrowhead or not via asocial or social learning, they click a HUNT button to see how many calories their arrowhead yields out of 1000. Their hunt score is added to their cumulative season score, and the participant is shown their rank relative to the five demonstrators. At the start of each season, participants' season scores are set to zero and the fitness functions are changed to new hidden values. In Seasons 1 and 2, fitness functions did not change during the 30 hunts. In Season 3, fitness functions changed to new random values without warning three times, on hunts 10, 15 and 23. Participants were informed that fitness functions did not change during Seasons 1 and 2, and may change during Season 3, but not on which hunts it would change.

After all three seasons were over, participants completed an on-screen individualism–collectivism questionnaire taken from the study of Sivadas *et al*. [33], rating their agreement on seven-point Likert scales to statements related to individualism (e.g. ‘I am a unique individual’) and collectivism (e.g. ‘If a co-worker gets a prize, I would feel proud’). The entire experiment took no more than 1 h to complete. UK and CI participants completed versions of the tasks in English (the latter had IELTS scores of more than 6.5). A Chinese version of the computer task was produced for the HK (traditional characters) and CM (simplified characters) participants using professional translators and was verified by the Hong Kong authors.

### (c) Design

Outcome variables are the frequency of social learning during a season (the proportion of the 29 hunts on which the participants chose to copy) and cumulative score at the end of the season (out of a maximum of 30 000 calories), for each of the three seasons. Predictor variables are culture (UK, CI, HK or CM), age, sex and measures of individualism and collectivism (analysed separately given evidence that they vary independently [34]). Quasi-binomial and linear regression analyses were conducted using the glm and lme commands in R v. 3.1.0 [35]. Quasi-binomial rather than binomial models were used for copying frequency data due to underdispersion [36] caused by many participants never copying.

## 3. Results

### (a) Cultural variation in copying frequency

**Figure 2.**
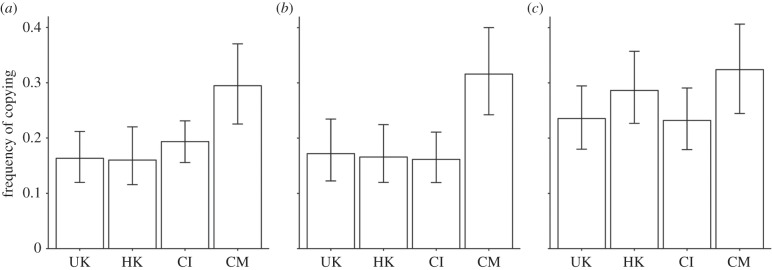
Mean copying frequencies for (*a*) Season 1 and (*b*) Season 2, which both featured no within-season environmental change and (*c*) Season 3, which featured within-season environmental change. UK, British; HK, Hong Kong; CI, Chinese immigrant; CM, Chinese mainland. Error bars show 95% CIs.

Both Seasons 1 and 2 showed similar patterns of copying, with CM participants copying more frequently than UK, HK and CI participants, who did not significantly differ (figure 2). Table 1 shows that for both Seasons 1 and 2 the best-fitting regression model retained culture and sex as significant predictors, with full models also containing age, individualism and collectivism showing no better fit than the culture-sex models (see the electronic supplementary material, tables S1–S3, for full model comparisons).

**Table 1.** Best-fitting regression models predicting copying frequency from culture and sex, separately for each season. Reference group for culture is UK, for sex is male. UK, British; HK, Hong Kong; CI, Chinese immigrant; CM, Chinese mainland. Models are quasi-binomial due to underdispersion in the data caused by several participants never copying.

Season	predictor	B	s.e.	*t*	*p*(>|*t*|)
1	(intercept)	−1.83	0.21	−8.84	<0.0001***
	HK	0.00	0.26	−0.01	0.99
	CI	0.15	0.25	0.60	0.55
	CM	0.77	0.23	3.31	0.0011**
	sex	0.36	0.18	2.03	0.0437*
2	(intercept)	−1.76	0.23	−7.81	<0.0001***
	HK	−0.02	0.28	−0.08	0.93
	CI	−0.13	0.29	−0.45	0.66
	CM	0.81	0.25	3.20	0.0016**
	sex	0.34	0.19	1.75	0.0812^†^
3	(intercept)	−1.18	0.17	−6.74	<0.0001***
	HK	0.26	0.24	1.09	0.28
	CI	−0.02	0.25	−0.08	0.94
	CM	0.44	0.24	1.86	0.0642^†^

Significance codes: ***<0.001, **<0.01, *<0.05, ^†^<0.1.

For Season 1, table 1 shows that the odds of a CM participant copying were e^0.77^ = 2.16 (95% CI[1.38, 3.42]) times the odds of a UK participant copying; HK and CI participants showed comparable copying frequencies to UK participants. For Season 2, the odds of a CM participant copying rose slightly to e^0.81^ = 2.25 (95% CI[1.38, 3.73]) times the odds of a UK participant copying, and HK and CI participants were again comparable to UK participants. The effect of sex was roughly half that of culture (table 1). The odds of a female participant copying in Season 1 was e^0.36^ = 1.43 (95% CI[1.01, 2.01]) times that of a male participant, and in Season 2 was e^0.34^ = 1.40 (95% CI[0.96, 2.06]) times that of a male participant.

The introduction of within-season environmental change in Season 3 revealed further cultural variation. While CM participants again copied more frequently than the other participants, the difference between CM and UK participants only approached significance (table 1), and a model including culture as a predictor did not fit the data significantly better than a null model (electronic supplementary material, table S3). As shown in figure 2, this is because the other cultural groups copied more frequently compared with previous seasons, bringing their copying up to near CM levels. Accordingly, Wilcoxon signed-rank tests comparing Season 2 versus 3 copying frequencies showed a significant increase in UK (*r* = 0.33, *p* < 0.001), HK (*r* = 0.42, *p* < 0.001) and CI (*r* = 0.21, *p* = 0.0129) participants and no change in CM (*r* = 0.04, *p* = 0.65) participants.

See the electronic supplementary material for analyses showing that participants were consistent in their social information use across seasons (electronic supplementary material, table S4) and predominantly employed payoff bias (electronic supplementary material, table S5), that CM participants copied more throughout the entirety of Seasons 1 and 2 (electronic supplementary material, figure S1), and a categorical breakdown of participants based on copying frequency (electronic supplementary material, table S6).

### (b) Relationship between copying frequency and score

Cumulative score at the end of each season is a measure of both performance within the game and real-world monetary payoff. To better understand the context of the cultural variation in copying frequency, we can ask whether copying is adaptive, i.e. led to higher scores/payoffs. Recall that each cultural group could learn from a different group of demonstrators, specific to their culture. Inspection of demonstrator scores (electronic supplementary material, table S7) shows that while they are on average similar across cultures, those of the highest-scoring demonstrator sometimes varied. Given that socially learning participants are employing payoff-bias and therefore selectively copying highest-scoring demonstrators, we must take this variation into account when assessing the adaptiveness of copying. We therefore calculated the *relative score* for each participant in each season, i.e. their cumulative score divided by the best demonstrator score for their cultural group. Relative scores less than 1 indicate that the participant performed worse than the best demonstrator, relative scores greater than 1 indicate superior performance to the best demonstrator.

**Figure 3.**
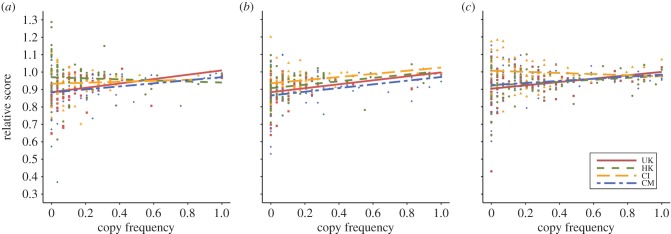
The relationship between frequency of copying and relative score for (*a*) Season 1, (*b*) Season 2 and (*c*) Season 3. Score is relative to the score of the best demonstrator in that cultural group; relative scores less than 1 indicate lower scores than the best demonstrator, greater than 1 indicate higher scores. Coloured lines and points show separate linear regressions for each culture. UK, British (red, squares); HK, Hong Kong (green, circles); CI, Chinese immigrant (orange, triangles); CM, Chinese mainland (blue, diamonds). (Online version in colour.)

Linear regression analyses (figure 3) show that, for Season 1, copying frequency significantly and positively predicts relative score for the UK (*b* = 0.0043, s.e. = 0.0016, *t*_74_ = 2.74, *p* = 0.0077) and CM (*b* = 0.0030, s.e. = 0.0014, *t*_71_ = 2.08, *p* = 0.0408) participants, but not CI (*b* = 0.0010, s.e. = 0.0018, *t*_68_ = 0.53, *p* = 0.60) or HK (*b* = −0.0010, s.e. = 0.0020, *t*_71_ = −0.50, *p* = 0.62) participants. As indicated in the electronic supplementary material, table S7, this is because the best demonstrator in the latter two groups performed no better than the average asocial learner in those groups. For Season 2, copying frequency significantly and positively predicts relative score for all four groups (UK: *b* = 0.0039, s.e. = 0.0011, *t*_74_ = 3.46, *p* = 0.0009; CM: *b* = 0.0036, s.e. = 0.0012, *t*_71_ = 3.13, *p* = 0.0025; CI: *b* = 0.0031, s.e. = 0.0015, *t*_68_ = 2.08, *p* = 0.0416; HK: *b* = 0.0034, s.e. = 0.0014, *t*_71_ = 2.41, *p* = 0.0185). For Season 3, copying significantly and positively predicts relative score in the UK participants (*b* = 0.0033, s.e. = 0.0013, *t*_74_ = 2.51, *p* = 0.0141), it approached significance in the CM (*b* = 0.0021, s.e. = 0.0012, *t*_71_ = 1.70, *p* = 0.0943) and HK (*b* = 0.0019, s.e. = 0.0012, *t*_71_ = 1.65, *p* = 0.10) participants, and CI participants showed no relationship (*b* = −0.0011, s.e. = 0.0014, *t*_68_ = −0.80, *p* = 0.43).

See the electronic supplementary material for analyses showing within-season changes in score (electronic supplementary material, figures S2 and S3) and analyses of absolute rather than relative scores (electronic supplementary material, figure S4 and table S7).

## 4. Discussion

Here we compared four cultural groups varying along an East–West continuum in their use of social versus asocial learning to solve a challenging task designed to reflect real-life learning about complex, cognitively opaque technological artefacts, typical of our species' cumulative culture. Unlike social psychology studies of cross-cultural variation in social learning [28], there was no participant-deception and no intuitively obvious solution. Unlike previous cultural evolution studies of the adaptiveness of social learning [10,15–22,31], we directly compared non-Western (CM) and Western (UK) samples using the same task and design, as well as intermediate Western-influenced (HK) and immigrant (CI) samples.

Throughout the first two seasons of hunting CM participants copied significantly more than UK, HK and CI participants. In order to maximize external validity, we ran different, culturally specific groups of demonstrators from whom experimental participants could learn. While this was more realistic than presenting identical and fictional demonstrators to all participants, it gave rise to unanticipated variation in the adaptiveness of social learning. In Season 1, social learning was adaptive relative to asocial learning in UK and CM participants because their highest-scoring demonstrators out-performed the average asocial learner, whereas HK and CI participants' highest-scoring demonstrators performed no better than the average asocial learner. We think it unlikely that these differences in demonstrator performance generated the observed differences in social learning, because (i) even if this explained the lower HK and CI copying, it could not explain the lower UK copying, and (ii) in neither HK or CI participants was there a significant *negative* relationship between score and copying, so even these participants could achieve the same score through frequent copying without the effort and risk of asocial learning. Moreover, (iii) in Season 2 all four cultural groups had highest-scoring demonstrators who out-performed the average asocial learner, resulting in a significant positive relationship between copying and score. Nevertheless, the same pattern of copying emerged as in Season 1, with CM participants copying roughly twice as often as the others.

We have therefore replicated, in our UK sample, the sub-optimal under-utilization of social information observed in the UK sample of a previous study that used the same task [15], and the Western samples of other studies that used different tasks [16–22]. Our finding that CM participants adaptively exploit social information to a greater extent suggests that Western sub-optimal underuse of social information may be part of broader cultural variation in learning strategies. Future cultural evolution experiments should pay greater attention to the cultural backgrounds of participants and use caution in generalizing findings to the entire species, a point that has been made for human behavioural studies in general [23]. We anticipate future studies going beyond the small number of specific populations that we studied here, and compiling a multi-population catalogue of social learning strategies used in diverse situations. We also found higher copying frequencies in female than male participants, a sex difference that has not been previously found using this task [15] but which deserves further examination.

Season 3 featured within-season environmental variation, which we predicted should reduce reliance on social learning given the risk of copying out-dated information [32]. Against expectations, not only did CM participants maintain their relatively high rates of copying, the other participants increased their copying frequencies to near CM levels. This increased copying may instead represent a ‘copy-when-uncertain’ social learning strategy, as found in previous experiments [17,18]. It also suggests that the cultural variation in copying observed in Seasons 1 and 2 is not fixed, and may change in response to task characteristics (albeit change in different ways in different cultures; it may be that Western or Westernized people are more responsive to changing conditions: [24]).

Despite their Chinese heritage, HK and CI participants were comparable to UK participants in their copying frequencies. We suggest that CI and HK participants have recently undergone a shift from Eastern ‘high social learning’ to Western ‘high asocial learning’ due to the increasing Westernization of China, especially in Hong Kong and the home cities of the CI participants (Shanghai, Beijing and Guangzhou), or, for the CI participants, direct Western influence from living in the UK. CM participants, coming from a relatively traditional and homogeneous region of China, have yet to experience this shift, although we might predict this in the coming decades with the increasing Westernization of China. Longitudinal studies tracking shifts in learning strategies in migrants as they move from East to West, or West to East, would provide a definitive test of this shift.

The presence of cultural variation in social information use, and potentially rapid changes in learning strategies in one generation or less, demands a greater understanding of the cultural processes underpinning learning strategies and the construction of models whereby learning strategies are themselves socially learned. Interestingly, recent studies suggest that social learning in non-human species may be influenced by individuals' early developmental cues [37] or past learning histories [38], echoing our conclusion. In humans, initial steps have been made to model the learning of learning strategies [29,30], but the full implications of this remain unexplored. This may shed light on exactly what ‘Westernization’ entails, and why it affects learning strategies in the way suggested by our results. Contrary to previous studies [17], individualism/collectivism was here unrelated to asocial/social learning (although these measures did not vary culturally in the expected manner: electronic supplementary material, figure S5). In any case, explanations in terms of individualism/collectivism simply beg the question of where variation in individualism/collectivism came from. Recent hypotheses for the origin of cultural variation in human learning and cognition include variation in historical rates of environmental change [24], subsistence practices [39] or pathogen prevalence [40]. A combination of theoretical models, laboratory experiments, historical data and longitudinal field studies are needed to further study the cultural (rather than genetic) evolution of learning strategies.

Social learning is thought to be key to understanding the uniqueness and evolutionary success of our species [11–14]. Such claims are often made by comparing learning strategies across species [11,12], and constructing theoretical models of the natural selection of genetically fixed learning strategies [4–6]. However, our finding of significant cultural variation in the frequency, adaptiveness and responsiveness of social learning suggests that there is no ‘species-typical’ pattern of social learning in humans (and potentially nor in other species), and no fixed genetic basis for learning strategies. Consequently, understanding human cultural evolution will require greater insight into how social processes such as migration, acculturation, education and globalization have created, and are currently changing, the means by which human culture is transmitted.

## Supplementary Material

Electronic Supplementary Material combined file

## Data accessibility

All experimental data generated in this study are available at Dryad doi:10.5061/dryad.f5q4s. Further details of methods and samples, further analyses and task screenshots are available in the electronic supplementary information.

## References

[1] Bandura A 1977 Social learning theory. Oxford, UK: Prentice-Hall.

[2] Durkheim E 1966 The rules of sociological method, 8th edn New York, NY: Free Press.

[3] Hoppitt W Laland KN 2013 Social learning: an introduction to mechanisms, methods, and models. Princeton, NJ: Princeton University Press.

[4] Rogers AR 1988 Does biology constrain culture? Am. Anthropol. 90, 819–831. (10.1525/aa.1988.90.4.02a00030)

[5] Boyd R Richerson PJ 1995 Why does culture increase human adaptability? Ethol. Sociobiol. 16, 125–143. (10.1016/0162-3095(94)00073-G)

[6] Enquist M Eriksson K Ghirlanda S 2007 Critical social learning: a solution to Rogers’ paradox of nonadaptive culture. Am. Anthropol. 109, 727–734. (10.1525/aa.2007.109.4.727)

[7] Lehmann L Feldman MW 2009 Coevolution of adaptive technology, maladaptive culture and population size in a producer–scrounger game. Proc. R. Soc. B 276, 3853–3862. (10.1098/rspb.2009.0724) PMC2817275 19692409

[8] Rendell L 2010 Why copy others? Insights from the social learning strategies tournament. Science 328, 208–213. (10.1126/science.1184719) 20378813 PMC2989663

[9] Rieucau G Giraldeau L-A 2011 Exploring the costs and benefits of social information use: an appraisal of current experimental evidence. Phil. Trans. R. Soc. B 366, 949–957. (10.1098/rstb.2010.0325) 21357217 PMC3049093

[10] Kameda T Nakanishi D 2003 Does social/cultural learning increase human adaptability? Rogers’ question revisited. Evol. Hum. Behav. 24, 242–260. (10.1016/S1090-5138(03)00015-1)

[11] Dean LG Kendal RL Schapiro SJ Thierry B Laland KN 2012 Identification of the social and cognitive processes underlying human cumulative culture. Science 335, 1114–1118. (10.1126/science.1213969) 22383851 PMC4676561

[12] Herrmann E Call J Hernandez-Lloreda MV Hare B Tomasello M 2007 Humans have evolved specialized skills of social cognition: the cultural intelligence hypothesis. Science 317, 1360–1366. (10.1126/science.1146282) 17823346

[13] Boyd R Richerson PJ Henrich J 2011 The cultural niche: why social learning is essential for human adaptation. Proc. Natl Acad. Sci. USA 108, 10 918–10 925. (10.1073/pnas.1100290108) PMC3131818 21690340

[14] Tennie C Call J Tomasello M 2009 Ratcheting up the ratchet: on the evolution of cumulative culture. Phil. Trans. R. Soc. B 364, 2405–2415. (10.1098/rstb.2009.0052) 19620111 PMC2865079

[15] Mesoudi A 2011 An experimental comparison of human social learning strategies: payoff-biased social learning is adaptive but underused. Evol. Hum. Behav. 32, 334–342. (10.1016/j.evolhumbehav.2010.12.001)

[16] Toelch U Bach DR Dolan RJ 2013 The neural underpinnings of an optimal exploitation of social information under uncertainty. Soc. Cogn. Affect. Neurosci. nst173v2 (10.1093/scan/nst173) PMC4221218 24194580

[17] Toelch U Bruce MJ Newson L Richerson PJ Reader SM 2014 Individual consistency and flexibility in human social information use. Proc. R. Soc. B 281, 20132864 (10.1098/rspb.2013.2864) PMC3871325 24352950

[18] Morgan TJH Rendell LE Ehn M Hoppitt W Laland KN 2011 The evolutionary basis of human social learning. Proc. R. Soc. B 279, 653–662. (10.1098/rspb.2011.1172) PMC3248730 21795267

[19] Eriksson K Strimling P 2009 Biases for acquiring information individually rather than socially. J. Evol. Psychol. 7, 309–329. (10.1556/JEP.7.2009.4.4)

[20] McElreath R Lubell M Richerson PJ Waring TM Baum W Edsten E Efferson C Paciotti B 2005 Applying evolutionary models to the laboratory study of social learning. Evol. Hum. Behav. 26, 483–508. (10.1016/j.evolhumbehav.2005.04.003)

[21] Efferson C Lalive R Richerson PJ McElreath R Lubell M 2008 Conformists and mavericks: the empirics of frequency-dependent cultural transmission. Evol. Hum. Behav. 29, 56–64. (10.1016/j.evolhumbehav.2007.08.003)

[22] Weizsacker G 2010 Do we follow others when we should? A simple test of rational expectations. Am. Econ. Rev. 100, 2340–2360. (10.1257/aer.100.5.2340)

[23] Henrich J Heine SJ Norenzayan A 2010 The weirdest people in the world? Behav. Brain Sci. 33, 61–135. (10.1017/S0140525X0999152X) 20550733

[24] Chang L Mak M Li T Wu BP Chen BB Lu HJ 2011 Cultural adaptations to environmental variability: an evolutionary account of east–west differences. Educ. Psychol. Rev. 23, 99–129. (10.1007/s10648-010-9149-0)

[25] Tweed RG Lehman DR 2002 Learning considered within a cultural context: Confucian and Socratic approaches. Am. Psychol. 57, 89–99. (10.1037/0003-066X.57.2.89) 11899565

[26] Yaveroglu IS Donthu N 2002 Cultural influences on the diffusion of new products. J. Int. Consum. Mark. 14, 49–63. (10.1300/J046v14n04_04)

[27] Triandis HC 1995 Individualism and collectivism. Boulder, CO: Westview Press.

[28] Bond R Smith PB 1996 Culture and conformity: a meta-analysis of studies using Asch's line judgment task. Psychol. Bull. 119, 111–137. (10.1037/0033-2909.119.1.111)

[29] Acerbi A Enquist M Ghirlanda S 2009 Cultural evolution and individual development of openness and conservatism. Proc. Natl Acad. Sci. USA 106, 18 931–18 935. (10.1073/pnas.0908889106) 19858478 PMC2776418

[30] Ghirlanda S Enquist M Nakamaru M 2006 Cultural evolution develops its own rules: the rise of conservatism and persuasion. Curr. Anthropol. 47, 1027–1034. (10.1086/508696)

[31] Efferson C Richerson P McElreath R Lubell M Edsten E Waring T Paciotti B Baum W 2007 Learning, productivity, and noise: an experimental study of cultural transmission on the Bolivian Altiplano. Evol. Hum. Behav. 28, 11–17. (10.1016/j.evolhumbehav.2006.05.005)

[32] Aoki K Wakano JY Feldman MW 2005 The emergence of social learning in a temporally changing environment: a theoretical model. Curr. Anthropol. 46, 334–340. (10.1086/428791)

[33] Sivadas E Bruvold NT Nelson MR 2008 A reduced version of the horizontal and vertical individualism and collectivism scale: a four-country assessment. J. Bus. Res. 61, 201–210. (10.1016/j.jbusres.2007.06.016)

[34] Oyserman D Coon HM Kemmelmeier M 2002 Rethinking individualism and collectivism: evaluation of theoretical assumptions and meta-analyses. Psychol. Bull. 128, 3–72. (10.1037/0033-2909.128.1.3) 11843547

[35] R Core Team. 2014 R: a language and environment for statistical computing. Vienna, Austria: R Foundation for Statistical Computing.

[36] Gelman A Hill J 2007 Data analysis using regression and multilevel/hierarchical models. New York, NY: Cambridge University Press.

[37] Lindeyer CM Meaney MJ Reader SM 2013 Early maternal care predicts reliance on social learning about food in adult rats. Dev. Psychobiol. 55, 168–175. (10.1002/dev.21009) 22315158

[38] Dawson EH Avarguès-Weber A Chittka L Leadbeater E 2013 Learning by observation emerges from simple associations in an insect model. Curr. Biol. 23, 727–730. (10.1016/j.cub.2013.03.035) 23562271

[39] Uskul AK Kitayama S Nisbett RE 2008 Ecocultural basis of cognition: farmers and fishermen are more holistic than herders. Proc. Natl Acad. Sci. USA 105, 8552–8556. (10.1073/pnas.0803874105) 18552175 PMC2438425

[40] Chiao JY Blizinsky KD 2010 Culture-gene coevolution of individualism/collectivism and the serotonin transporter gene. Proc. R. Soc. B 277, 529–537. (10.1098/rspb.2009.1650) PMC2842692 19864286

