# Supplementary material for: Higher frequency of social learning in China than in the West shows cultural variation in the dynamics of cultural evolution
Source: Proc Biol Sci. 2015 Jan 7;282(1798):20142209. doi: 10.1098/rspb.2014.2209 (PMC4262178; doi:10.1098/rspb.2014.2209)
Supplement: Electronic Supplementary Material combined file [file rspb20142209supp1.pdf]

Electronic Supplementary Material for:

Higher frequency of social learning in China than in the West shows cultural variation in the dynamics of cultural evolution

Alex Mesoudi, Lei Chang, Keelin Murray, Hui Jing Lu

Contents:

1. Additional details of participant samples

2. Additional statistical analyses

3. Screenshots of the task

1. Additional details of participant samples

1.1 Education / intellectual ability / confidence

An alternative interpretation of our finding that Chinese Mainland (CM) participants copied more frequently than the other samples is that the CM participants were lower in intellectual ability, academic experience, or confidence than the British (UK), Chinese Immigrant (CI) and Hong Kong (HK) participants, therefore leading to their higher rates of social learning beyond any broader cultural differences.

We think this unlikely for three reasons. First, the two Chinese universities (Hong Kong Polytechnic and Chao Zhou Normal University) were specifically selected to be as comparable as possible. HK Poly is ranked 5th out of 8 universities in Hong Kong, and is colloquially classed as ‘third-tier’ in having regional but not national or international standing (first tier would be internationally known, e.g. Harvard or Cambridge; second tier would be nationally known, e.g. Rutgers or Liverpool; third tier would be regionally known, e.g. Cal State or Teeside). Chao Zhou NU is ranked 2nd out of 3 universities in the Chao Zhou region, and 546th out of 1079 in China (http://www.gxeduw.com/gaokao/201379354.html), also classing as third-tier. Regionally, Hong Kong and Chao Zhou are comparable in educational level: both have compulsory secondary education, with just a slightly higher proportion (38%) of Hong Kong high school graduates going on to university than Chao Zhou high school graduates (32%). Consequently, intellectual abilities and confidence are expected to be very similar between these two samples, and given that the HK participants copied significantly less than the CM participants throughout Seasons 1 and 2, this is unlikely to be the cause of the difference.

Second, while the UK and CI participants were studying at Durham University, a top-tier university (typically ranked 5th out of 123 in the UK: www.thecompleteuniversityguide.co.uk), the UK participants here performed comparably to Queen Mary students in a previous experiment using the same task [1] (see ESM Section 2.6). Queen Mary is ranked 36th out of 123 in the UK, with the sample in that study coming predominantly from the School of Biological and Chemical Sciences, ranked 37th out of 99. Queen Mary is probably borderline second/third tier by the above definitions, and has lower entry requirements than Durham, so the fact that our Durham UK sample performed identically to the previous Queen Mary UK sample again suggests that intelligence/confidence is unlikely to be responsible for the observed differences in the present study.

Third, table S7 shows the performance of the asocial-learning-only demonstrators, while table S6 shows the performance of experimental participants who chose not to copy (effectively making them asocial-learning-only participants as well). If the CM group were less intellectually able, less confident, or less familiar with computer tasks, then we would expect the CM asocial-

learners to perform less well than asocial learners from the other cultural groups. In contrast, tables S6 and S7 show no significant differences between the demonstrators in overall performance. In fact, in both Season 1 and 2 the CM demonstrators had the highest mean and maximum scores.

1.2 Language differences

The UK and CI participants conducted the task in English (figure 1*a*) while the HK and CM participants conducted the task in Cantonese (figure 1*b*). While the CI participants were unique in conducting the experiment in their second language, all international students at Durham are required to demonstrate their English reading and writing proficiency upon entry (specifically, an IELTS overall score of 6.5 or more). The fact that the UK and CI participants performed identically in the task suggests that this was no impediment to the CI participants' understanding or performance.

The HK participants saw the task in traditional Chinese characters, while the CM participants saw the task in simplified Chinese characters. These are two alternative systems for writing the Chinese language as learned in school: traditional characters are standard in Hong Kong, while simplified characters are standard in mainland China. While our HK participants may have been able to have understood simplified characters, it would have been unusual to have been presented with a task in simplified characters. It is unlikely that our CM participants would have been able to understand traditional characters. This difference was therefore unavoidable. In any case, given that HK and CM participants were each using their standard mode of writing (equivalent to the UK participants using English), we think it unlikely that the use of these different character sets was responsible for the differences observed between the CM and HK participants.

Instructions were translated and standardised across participants (see ESM section 3 below). The task was administered by author KM in the UK and CI participants, and author HJL in the HK and CM participants.

1.3 Knowledge of other groups / demonstrator differences

As noted in the main text, experimental participants could copy one of a set of demonstrators from their own cultural background, i.e. UK participants could copy UK demonstrators, HK participants could copy HK demonstrators, and so on. This was done so as to avoid potentially unusual and (for our purposes) irrelevant ingroup-outgroup effects, such that participants from one group may have copied participants from a perceived outgroup differently to how they would have copied ingroup members. The latter was our interest here, given that the most common interactions are with ingroup members. Participants were informed that demonstrators had been recruited in the same

way as they had been, and so were of the same cultural background. We avoided deception by actually running these demonstrator groups in the four samples, rather than using the same demonstrators for all participants and labelling them differently. While this created some differences in the adaptiveness of social learning in Seasons 1 and 3 (see main text), we think that this loss of internal validity is worth the cost of an increase in external validity. Future experiments might more systematically explore the reactions of participants to demonstrators of different ability and cultural background. Finally, participants were unaware that the experiment was part of a cross-cultural study, so as to avoid them second-guessing how they thought they should behave in comparison to participants from another culture.

2. Additional statistical analyses

2.1 Model comparison for Season 1 copying frequency

Table S1 compares the null (intercept-only) model, culture-only model, culture+sex model (presented in the main text in table 1), and a full model with all predictors, for the dependent measure Season 1 copying frequency. The culture-only model showed significantly better fit to the data than the null model (likelihood ratio test: deviance=5.15, *df*=3, *p*=0.0016); adding sex as a predictor improved model fit further (likelihood ratio test: deviance=1.39, *df*=1, *p*=0.0413). The full model did not significantly improve model fit compared to the culture-sex model (likelihood ratio test: deviance=0.42, *df*=3, *p*=0.74), hence the culture-sex model is reported in the main text. Subsequent post-hoc pairwise Tukey contrasts on the culture+sex model confirmed that the CM participants also significantly differed from the HK (*b*=0.77, *SE*=0.24, *z*=3.26, *p*=0.0060) and CI (*b*=0.62, *SE*=0.23, *z*=2.67, *p*=0.0380) participants, and that the CI and HK participants did not differ (*b*=0.15, SE=0.26, *z*=0.60, *p*=0.93). Neither age, individualism or collectivism were significant predictors alone, and nor were there any interactions between any of the predictors.

Table S1. Model comparison for Season 1 copying frequency. Reference group for culture is UK, for sex is male. Quasibinomial regression was used due to underdispersal in the data caused by many participants never copying (dispersion parameter for null model=0.36, for culture-only model=0.34, culture+sex model=0.33). Residual deviance is reported because AIC and DIC are inappropriate for quasibinomial models. Significance codes: ***<0.001, **<0.01, *<0.05.

Model	Residual deviance	df	Predictor	B	SE	t	p(>|t|)
Null	102.64	291	(Intercept)	-1.37	0.09	-15.69	<0.0001***
Culture	97.49	288	(Intercept)	-1.63	0.18	-9.06	<0.0001***
			Culture=HK	-0.02	0.26	-0.09	0.93
			Culture=CI	0.21	0.25	0.82	0.41
			Culture=CM	0.76	0.23	3.25	0.00129**
Culture + sex	96.10	287	(Intercept)	-1.83	0.21	-8.84	<0.0001***
			Culture=HK	-0.00	0.26	-0.01	0.99
			Culture=CI	0.15	0.25	0.60	0.55
			Culture=CM	0.77	0.23	3.31	0.0011**
			Sex=female	0.36	0.18	2.03	0.0437*
Full	95.69	284	(Intercept)	-0.71	1.15	-0.61	0.54
			Culture=HK	0.02	0.26	0.07	0.95
			Culture=CI	0.19	0.26	0.74	0.46
			Culture=CM	0.84	0.24	3.44	0.0007***
			Age	-0.04	0.05	-0.89	0.37
			Sex=female	0.30	0.18	1.63	0.10
			Individualism	-0.08	0.13	-0.63	0.53
			Collectivism	0.02	0.12	0.15	0.88

2.2 Model comparison for Season 2 copying frequency

Table S2 compares the null (intercept-only) model, culture-only model, culture+sex model (presented in the main text in table 1) and a full model with all predictors, for the dependent measure Season 2 copying frequency. The culture-only model showed significantly better fit to the data than the null model (likelihood ratio test: deviance=7.06, df=3, p=0.0007); adding sex as a predictor improved model fit further, albeit not quite reaching significance at p<0.05 (likelihood ratio test: deviance=1.27, df=1, p=0.0783). The full model did not significantly improve model fit compared to the culture+sex model (likelihood ratio test: deviance=0.49, df=3, p=0.75), hence the culture-sex model is reported in the main text. Subsequent post-hoc pairwise Tukey contrasts on the culture+sex model confirmed that the CM participants also significantly differed from the HK (b=0.83, SE=0.26, z=3.22, p=0.0070) and CI (b=0.94, SE=0.27, z=3.52, p=0.0027) participants, and that the CI and HK participants did not differ (b=0.10, SE=0.29, z=0.36, p=0.98). Neither age, individualism or collectivism were significant predictors alone, and nor were there any interactions between any of the predictors.

Table S2. Model comparison for Season 2 copying frequency. Reference group for culture is UK, for sex is male. Quasibinomial regression was used due to underdispersal in the data caused by many participants never copying (dispersion parameter for null model=0.44, for culture-only model=0.41, culture+sex model=0.41). Residual deviance is reported because AIC and DIC are inappropriate for quasibinomial models. Significance codes: ***<0.001, **<0.01, *<0.05, †<0.1.

Model	Residual deviance	df	Predictor	B	SE	t	p(>|t|)
Null	126.05	291	(Intercept)	-1.36	0.10	-14.16	<0.0001***
Culture	119.00	288	(Intercept)	-1.57	0.20	-8.04	<0.0001***
			Culture=HK	-0.04	0.28	-0.16	0.88
			Culture=CI	-0.07	0.29	-0.26	0.79
			Culture=CM	0.80	0.25	3.15	0.0018**
Culture + sex	117.72	287	(Intercept)	-1.76	0.23	-7.81	<0.0001***
			Culture=HK	-0.02	0.28	-0.08	0.93
			Culture=CI	-0.13	0.29	-0.45	0.66
			Culture=CM	0.81	0.25	3.20	0.0016**
			Sex=female	0.34	0.19	1.75	0.0812†
Full	117.23	284	(Intercept)	-1.69	1.28	-1.32	0.19
			Culture=HK	-0.07	0.28	-0.24	0.81
			Culture=CI	-0.16	0.29	-0.54	0.59
			Culture=CM	0.79	0.26	3.02	0.0028**
			Age	-0.01	0.05	-0.22	0.83
			Sex=female	0.38	0.20	1.86	0.0639†
			Individualism	0.13	0.15	0.85	0.40
			Collectivism	-0.10	0.13	-0.78	0.44

2.3 Model comparison for Season 3 copying frequency

Table S3 compares the null (intercept-only) model, culture model (presented in the main text in table 1) and a full model with all predictors, for the dependent measure Season 3 copying frequency. As in Seasons 1 and 2, CM participants' copying frequency was higher than the other participants', although it only approached significance at $p<0.05$. Neither the culture-only model (likelihood ratio test: deviance=2.14, df=3, p=0.16) nor the full model (likelihood ratio test: deviance=2.51, df=7, p=0.55) significantly improved model fit compared to the null. Neither age, sex, individualism or collectivism were significant predictors alone, and nor were there any interactions between any of the predictors.

Table S3. Model comparison for Season 3 copying frequency. Reference group for culture is UK, for sex is male. Quasibinomial regression was used due to underdispersal caused by many participants never copying (dispersion parameter for null model=0.42, for culture-only model=0.42). Residual deviance is reported because AIC and DIC are inappropriate for quasibinomial models. Significance codes: ***<0.001, **<0.01, *<0.05, †<0.1.

Model	Residual deviance	df	Predictor	B	SE	t	p(>|t|)
Null	135.04	291	(Intercept)	-1.00	0.09	-11.62	<0.0001***
Culture	132.90	288	(Intercept)	-1.18	0.17	-6.74	<0.0001***
			Culture=HK	0.26	0.24	1.09	0.2763
			Culture=CI	-0.02	0.25	-0.08	0.9392
			Culture=CM	0.44	0.24	1.86	0.0642†
Full	132.53	284	(Intercept)	-1.01	1.15	-0.88	0.3777
			Culture=HK	0.24	0.25	0.98	0.3261
			Culture=CI	-0.05	0.26	-0.20	0.8456
			Culture=CM	0.43	0.25	1.75	0.0816†
			Age	-0.03	0.04	-0.58	0.5599
			Sex=female	0.05	0.18	0.28	0.7774
			Individualism	0.09	0.14	0.68	0.4952
			Collectivism	-0.01	0.12	-0.12	0.9067

2.4 Consistency of social information use and score within participants across seasons

Table S4 shows the correlations across seasons within participants in copying frequency (top right correlations) and cumulative season score (bottom left correlations). Overall there is high within-participant consistency in both copying frequency and score. Correlations are higher for copying frequency, which is to be expected given that copying was directly under the participants' control, whereas score was also affected by asocial learning (e.g. whether they found one of the high-fitness arrowhead designs).

Table S4. Within-participant consistency across seasons in copying frequency (top right cells) and score (bottom left cells). Values are Spearman's rank correlations r_s. All correlations are significant at $p<0.001$***.

	Season 1	Season 2	Season 3
**Season 1**	-	0.69***	0.59***
**Season 2**	0.42***	-	0.70***
**Season 3**	0.27***	0.30***	-

2.5 Use of payoff-biased social learning

Table S5 shows the frequency with which participants copied the best demonstrator, as opposed to copying any of the five demonstrators. Copy-best-demonstrator frequencies were consistently 70-80% as high as the overall copying frequencies, indicating use of a payoff-biased social learning strategy. If participants were copying at random, we would expect these frequencies to be one-fifth (20%) of the overall copying frequencies. Regression analyses conducted on copy-best-demonstrator frequencies yielded qualitatively identical results as the regression analyses reported in the main text which used overall copying frequencies.

Table S5. Proportion of hunts on which participants chose to copy one of the demonstrators ('Copying frequency'), and proportion of hunts on which participants chose to copy the *most successful* demonstrator, defined as the demonstrator with the highest cumulative score on that hunt ('Copy-best-demonstrator frequency'), broken down by Season and Culture. Values are proportions across all hunts and participants ± 95% confidence intervals. The 'All' column shows the overall frequency across all participants. UK=British, HK=Hong Kong, CI=Chinese Immigrant, CM=Chinese Mainland.

		UK	HK	CI	CM	All
**Copying frequency**	**Season 1**	0.16±0.05	0.16±0.05	0.19±0.04	0.29±0.08	0.20±0.03
	**Season 2**	0.17±0.06	0.17±0.05	0.16±0.04	0.32±0.08	0.20±0.03
	**Season 3**	0.24±0.06	0.29±0.07	0.23±0.06	0.32±0.08	0.27±0.03
**Copy-best-demonstrator frequency**	**Season 1**	0.14±0.04	0.11±0.04	0.17±0.04	0.23±0.07	0.16±0.02
	**Season 2**	0.15±0.05	0.13±0.05	0.14±0.04	0.22±0.06	0.16±0.02
	**Season 3**	0.19±0.05	0.20±0.05	0.18±0.05	0.24±0.06	0.20±0.03

2.6 Comparison with copying frequency found in previous study

The copying frequency seen in the UK participants of the present study for Season 1 (mean=0.16, SE=0.02) and Season 2 (mean=0.17, SE=0.03) shown in Table S5 is slightly lower than the frequency of copying in UK participants from a previous study [1] that used the same task and a

similar design (mean=0.23, SE=0.04; see Table 1 of reference [1]). The latter is, however, lower than the copying frequency for CM participants in the present study (Season 1: mean=0.29, SE=0.04; Season 2: mean=0.32, SE=0.04). Note that [1] had a different design where participants could employ multiple social learning strategies, including conformity, random copying and averaging as well as payoff-bias, which may have inflated the copying frequency. Focusing only on payoff-biased social learning in the previous study (the predominant strategy used in the present study; see Section 2.5) gives a more comparable copying frequency (mean=0.19, SE=0.04) to our UK participants. Overall, the similarity between the two UK samples increases our confidence in the validity of the present study's findings and methods.

2.7 Within-season changes in copying frequency

Figure S1 shows within-season changes in copying frequency per hunt for the four cultures. Figure S1 clearly shows the CM participants consistently copying more often than the other participants throughout Seasons 1 and 2. The other groups, particularly the CI participants, tend to copy more frequently at the beginning of the seasons. In Season 3, the CM participants start out copying more as in previous seasons, but following the first environmental shift on Hunt 10 the other cultural groups increased their copying frequency. Interestingly, by the final few hunts of Season 3, the CM participants were again copying the most. Note also that participants did not seem to change their initial frequency of copying at the start of Season 3 in response to being told that the environment may change; it was only after the first shift on Hunt 10, and particularly the big shift on Hunt 15 (see below), that the non-CM participants increased their copying.

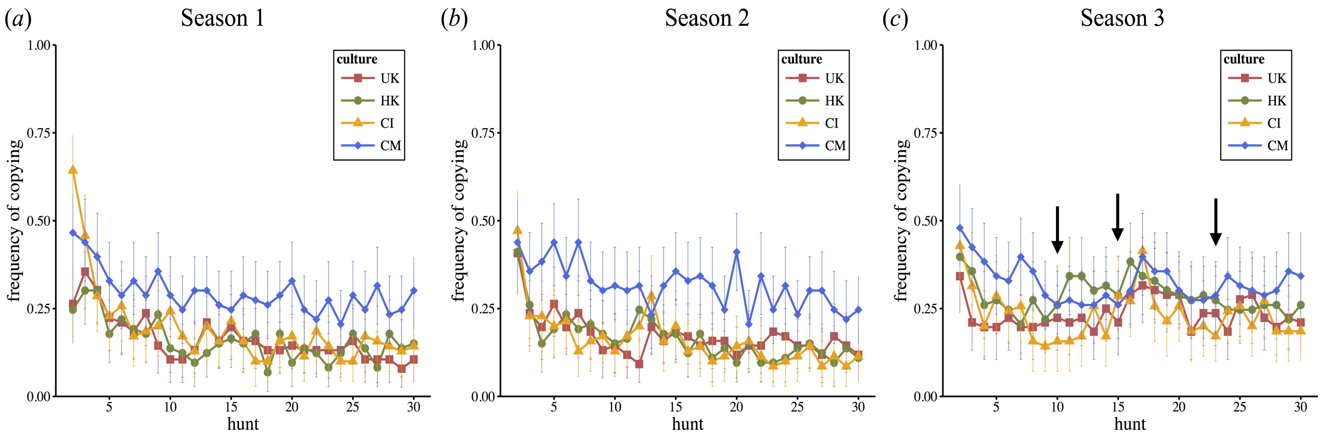

Figure S1. Within-season change in mean copying frequency per hunt (i.e. the proportion of participants who copied on that hunt) for (*a*) Season 1, (*b*) Season 2 and (*c*) Season 3. Coloured lines indicate different cultural groups: UK=British, HK=Hong Kong, CI=Chinese Immigrant, CM=Chinese Mainland. Error bars show 95% confidence intervals. Black arrows indicate environmental shifts in Season 3.

2.8 Categorical breakdown of participants based on copying frequency

Table S6 shows the numbers and mean scores of participants broken down into categories by their copying frequency: zero-copiers who never copied, low-copiers who copied between 1 and 10 times (out of 29), and high copiers who copied between 11 and 29 times (out of 29). The numbers reflect the regression analyses presented above: (i) there are more than twice as many CM high-copiers in Seasons 1 and 2 than there are UK, HK and CI high-copiers, reflecting the regressions for copying frequency; (ii) there is a similar number of CM high copiers in Season 3 than there were CM copiers for Seasons 1 and 2, but in Season 3 the other cultural groups have more high copiers; (iii) in general, high copiers out-perform low- and zero-copiers, particularly for Season 2 when there is a strong relationship between score and copying (see figure 3).

Table S6. Numbers and mean scores for different categories of copiers. Zero-copiers never copied on any hunt, low-copiers copied between one and ten times, high-copiers copied between 11 and 30 times.

		Number of participants			Mean score		
		**Zero copiers**	**Low copiers**	**High copiers**	**Zero copiers**	**Low copiers**	**High copiers**
**Season 1**	**UK**	11	58	7	20787	22830	23645
	**HK**	19	46	8	22402	21040	20684
	**CI**	7	53	10	21660	21233	21926
	**CM**	17	34	22	23305	23391	24613
**Season 2**	**UK**	24	42	10	21786	23249	24251
	**HK**	24	38	11	21399	21949	22624
	**CI**	17	46	7	22191	23294	23792
	**CM**	19	31	23	21131	23428	24144
**Season 3**	**UK**	17	41	18	18255	20049	20058
	**HK**	15	35	23	18239	18557	18951
	**CI**	11	47	12	19572	18786	18953
	**CM**	18	31	24	17645	18948	19216

2.9 Comparison of demonstrator performance

Table S7 shows descriptive statistics for the four five-person groups of asocial demonstrators, from whom experimental participants could copy throughout the task. Overall, Kruskal-Wallis tests showed that there are no differences in score between the cultural groups for Season 1 ($X^2(3)=3.86$, $p=0.28$), Season 2 ($X^2(3)=0.71, p=0.87$) or Season 3 ($X^2(3)=2.29, p=0.51$). However, given that

participants are employing payoff-bias and preferentially copying the most successful demonstrator (see Table S5), it is more relevant to look at the maximum demonstrator score (final column of Table S7). Although there are too few data points to conduct statistical comparisons, we can see that in Season 1 the best CM demonstrator achieved a much higher score (26,114) than the best demonstrator of the other groups, particularly the HK (22,123) and CI (22,765) best-demonstrators. Indeed, the latter scores are not much different to the mean score of zero-copiers (asocial learners) of those groups shown in Table S6. The best-demonstrator performance is more comparable in Season 2, with best demonstrators out-performing mean asocial learners across all cultures. In Season 3 the CI best-demonstrator (18,951) scores lower than the other groups, and indeed the CI mean zero-copier score (19,572), hence the negatively-sloped regression line in figure 3. Because participants' absolute scores are differentially affected by the different best-demonstrator scores, in the analysis presented in the main text we weighted participants' scores by their best demonstrator score. This does, however, mean that scores are not comparable across cultures, only within each culture.

Table S7. Descriptive statistics for the scores of the four five-person groups of asocial-learning-only demonstrators, for each season and culture.

Season	Culture	Mean	SD	Min	Max
1	UK	21652	3851	15032	24974
	HK	21446	433	20913	22123
	CI	20640	1370	19270	22765
	CM	21798	4662	13961	26114
2	UK	21528	2849	17467	25381
	HK	21029	2574	18339	23691
	CI	22024	1602	20217	24308
	CM	21858	4008	15092	25665
3	UK	19103	1964	16484	21214
	HK	18093	1379	16235	19856
	CI	17024	2033	13773	18951
	CM	17938	1456	16365	19828

2.10 Within-season changes in score

Figure S2 shows that CM participants out-performed the other participants from the first hunt of Season 1. The UK participants eventually caught up with them in terms of score-per-hunt, but as the regression analyses in Section 2.11 show, this was not enough to exceed the CM participants' cumulative season score. In Season 2, all cultural groups performed roughly equally well, although the CM participants finished slightly higher than the other groups. In Season 3 we can see the effect of the environmental shifts on hunts 10, 15 and 23; the shift on hunt 15 appeared to be particularly detrimental to scores (the new values following the shift were randomly chosen, although were the same for all participants in all groups).

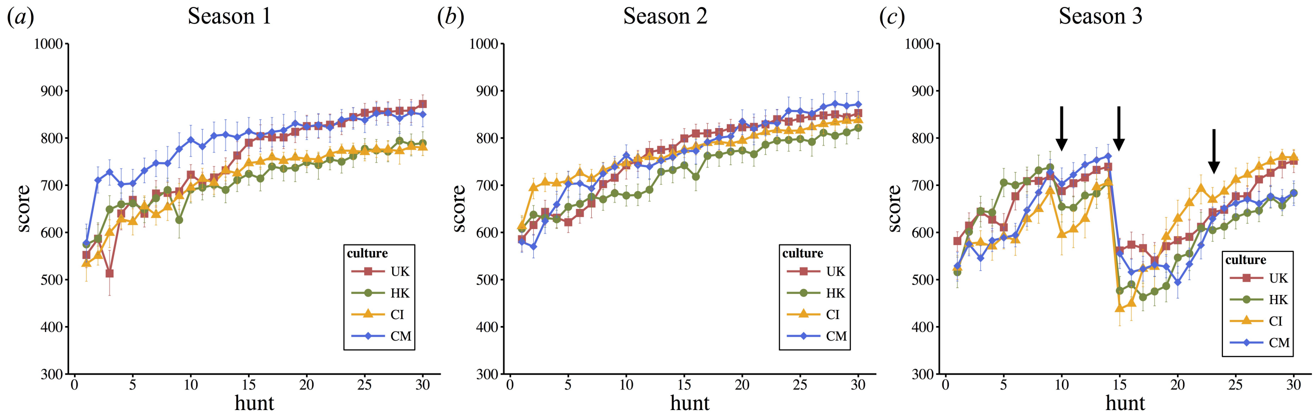

Figure S2. Within-season change in mean score (in calories, maximum per hunt = 1000) for (*a*) Season 1, (*b*) Season 2 and (*c*) Season 3. Coloured lines indicate different cultural groups: UK=British, HK=Hong Kong, CI=Chinese Immigrant, CM=Chinese Mainland. Error bars show 95% confidence intervals. Black arrows indicate environmental shifts in Season 3.

Figure S3 shows mean score within each season, but with participants divided into the copying categories shown in Table S6. First, note that the zero-copiers in all three seasons improved their scores over the season, indicating that even these pure-asocial learners understood the task and successfully engaged in asocial learning to improve their arrowheads. Second, note that in Season 1, Season 2, and Season 3 before the environmental shift, high-copiers (who copied on 11 or more of the hunts) out-performed the low- and zero-copiers. Low-copiers outperformed zero-copiers in the latter part of Season 1, and throughout Season 2. Finally, it is interesting that high-copiers took the longest to recover from the big hunt-15 environmental shift in Season 3, although they quickly recovered to out-perform zero-copiers.

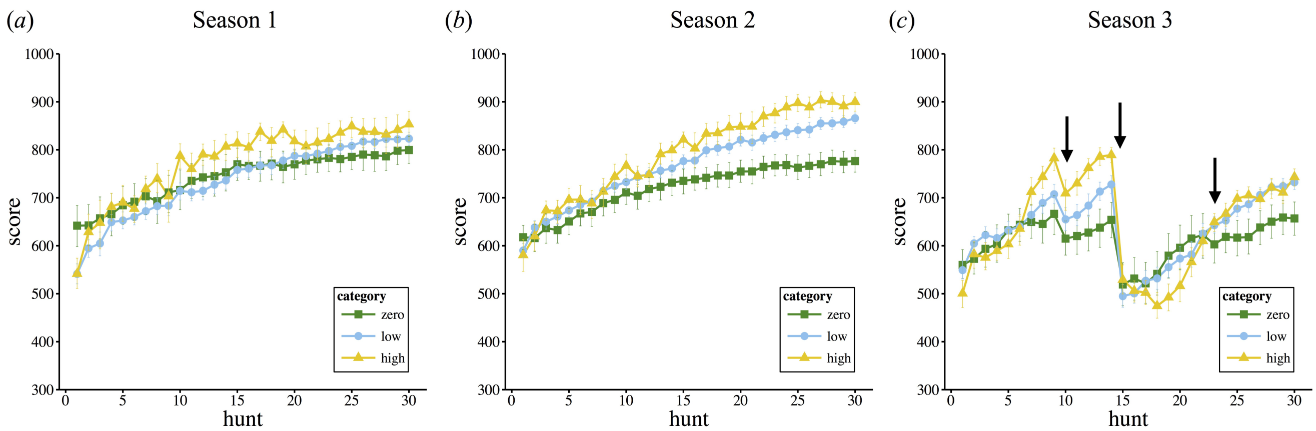

Figure S3. Within-season change in mean score (in calories, maximum per hunt = 1000) for (*a*) Season 1, (*b*) Season 2 and (*c*) Season 3. Coloured lines indicate different copying categories (see Table S8): ‘zero’ = never copied, ‘low’ = copied between 1 and 10 times, ‘high’ = copied between 11 and 29 times. Error bars show 95% confidence intervals. Black arrows indicate environmental shifts in Season 3.

2.11 Regression analyses of absolute score

Figure S4 shows the relationship between absolute score and frequency of copying for the three seasons. Absolute score is the actual cumulative score achieved by participants within the task, although as noted above and in the main text, this is differentially influenced by best-demonstrator scores so relative score is used in the main text. Note that the regression lines in figure S5 have identical slopes to those in figure 3, they are just have different intercepts. The thick black line shows the significant overall regression across all participants with culture as a random effect (see table S7). Coloured lines and points show separate regression lines and data for the four cultures.

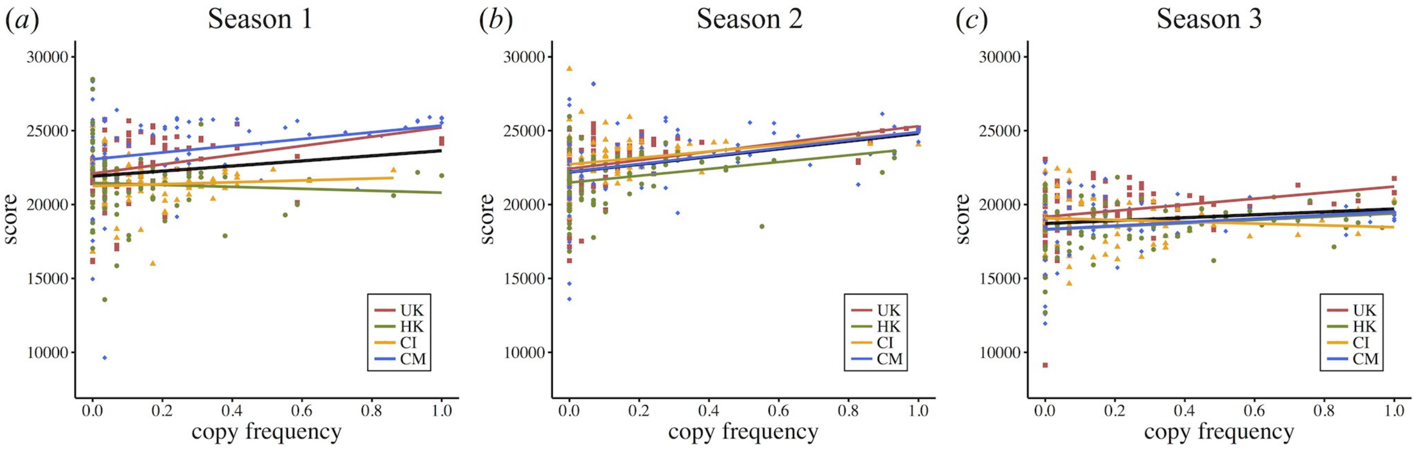

Figure S4. The relationship between frequency of copying and absolute score for (*a*) Season 1, (*b*) Season 2, and (*c*) Season 3. The thick black line shows the overall regression line with culture as a random factor. Coloured lines and points show separate regressions for each culture.

Table S7. Model comparison for multilevel model predicting score from copy frequency. For each season, Model 1 contains no multilevel structure nor predictors, Model 2 adds varying intercepts by culture, and Model 3 adds copy frequency as a predictor. In each case model fit significantly improves. Significance codes: ***<0.001, **<0.01, *<0.05.

**Season**	**Model**	**AIC**	**Log Likelihood**	**Test**	**Likelihood Ratio**	**p value**
1	1. Null	5414	-2705			
	2. Varying intercepts	5383	-2689	1 vs. 2	32.87	<0.0001***
	3. Copy frequency	5377	-2684	2 vs. 3	8.54	0.0035**
2	1. Null	5311	-2653			
	2. Varying intercepts	5306	-2650	1 vs. 2	6.64	0.01**
	3. Copy frequency	5276	-2634	2 vs. 3	32.65	<0.0001***
3	1. Null	5226	-2610.78			
	2. Varying intercepts	5222	-2608.03	1 vs. 2	5.51	0.0190*
	3. Copy frequency	5217	-2604.46	2 vs. 3	7.13	0.0076**

For Season 1 (figure S4*a*), there was an overall positive and significant relationship between absolute score and copying frequency (b=59.28, SE=20.17, t(287)=2.94, p=0.0036). Linear regression models for each culture separately showed a significant effect of copy frequency in the UK (b=107.40, SE=39.19, t=2.74, p=0.0077) and CM (b=78.65, SE=37.75, t=2.08, p=0.0408) participants but not the HK (b=-22.68, SE=45.14, t=-0.50, p=0.62) and CI (b=21.97, SE=41.51, t=0.53, p=0.60) participants.

For Season 2 (figure S4*b*), there was a stronger overall relationship between score and frequency of copying (b=90.21, SE=15.40, t(287)=5.86, p<0.0001). However, a comparison of regression coefficients using the method provided in [2] showed that the relationship for Season 2 was not significantly different to that for Season 1 (z=1.22, p=0.22). Linear regression models showed a significant effect of copy frequency in the UK (b=99.37, SE=28.75, t=3.46, p=0.0009), CM (b=93.54, SE=29.88, t=3.13, p=0.0025), HK (b=79.87, SE=33.12, t=2.41, p=0.0185) and CI (b=75.05, SE=36.13, t=2.08, p=0.0416) participants.

For Season 3 (figure S4*c*), there was a weaker overall relationship between score and frequency of copying (b=34.01, SE=12.66, t(287)=2.69, p<0.008). A comparison of regression coefficients using the method provided in [2] showed that the relationship for Season 3 was not significantly different to that for Season 1 (z=1.06, p=0.29), but was significantly lower than for

Season 2 (z=2.82, p=0.0048). Linear regression models showed a significant effect of copy frequency in the UK group (b=70.59, SE=28.09, t=2.51, p=0.014) but not the other groups (CI: b=-21.12, SE=26.44, t=-0.80, p=0.427; CM: b=41.05, SE=24.20, t=1.70, p=0.094; HK: b=37.83, SE=22.97, t=1.65, p=0.104). Comparisons between the regression coefficients of the different cultures using the same method showed that only the UK and CI lines significantly differed (z=2.38, p=0.017).

2.12 Cultural variation in individualism and collectivism

Figure S5 shows cultural variation in (a) collectivism and (b) individualism. Neither measure showed the expected pattern of higher individualism and lower collectivism in the Western UK group, lower individualism and higher collectivism in the East Asian CM group, and CI and HK groups either equivalent to the CM group or intermediate between UK and CM groups.

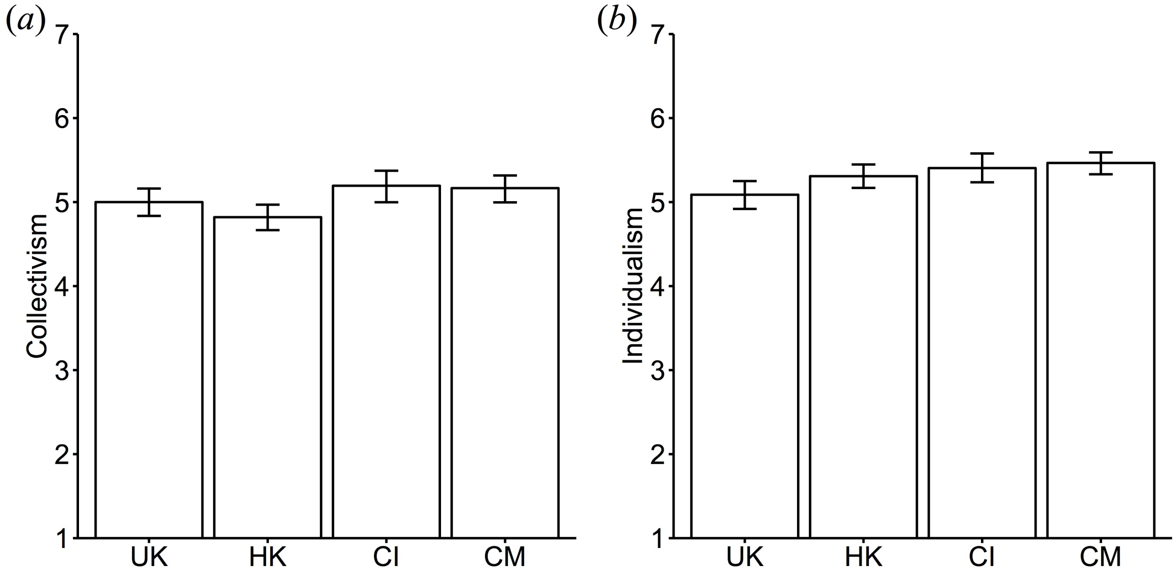

Figure S5. Cultural variation in (*a*) collectivism and (*b*) individualism across the four cultural groups, as measured using the 14-item scale developed by [3]. Scores are mean ratings for agreement with seven collectivism statements or seven individualism statements, all on 7-point Likert scales. Error bars show 95% confidence intervals.

To determine whether cultural groups differed in collectivism, linear regression was used with collectivism as the dependent measure and culture as a predictor. A model with culture as a predictor showed significantly better fit compared to a no-predictor null model (likelihood ratio test: deviance=6.48, df=3, p=0.0076). Tukey post-hoc contrasts revealed that this was because the HK participants were significantly less collectivist than the CI (b=0.37, SE=0.12, z=3.04, p=0.0125) and CM (b=0.35, SE=0.12, z=2.84, p=0.0236) participants, with no other contrasts significant. Similarly,

linear regression with individualism as the dependent measure also showed significantly better fit with culture as a predictor compared to a no-predictor null model (likelihood ratio test: deviance=6.15, *df*=3, *p*=0.0033). Tukey post-hoc contrasts revealed that this was because the UK group was significantly less individualist than the CI (*b*=0.32, *SE*=0.11, *z*=2.86, *p*=0.022) and CM (*b*=0.38, *SE*=0.12, *z*=3.44, *p*=0.0031) participants, with no other contrasts significant.

These unexpected findings suggest that this questionnaire may not be a suitable tool for measuring cross-cultural variation in social orientation and explain why individualism and collectivism were not significant predictors of copying frequency (see main text). This may be because (i) we used a reduced and potentially less reliable questionnaire compared to more commonly-used measures of individualism and collectivism, (ii) there was some kind of priming effect of the experimental task, or (iii) our groups genuinely do not vary in individualism-collectivism. Further studies would be needed to distinguish between these possibilities.

3. Screenshots of the task

Figure S6 shows screenshots of the task. (*a*) to (*h*) contain instructions explaining the task and procedure, before the five-hunt asocial-learning-only practice session shown in (*i*). (*j*) to (*l*) provide further instructions before the main task shown in (*m*) (and Figure 1). (*n*) is shown to participants immediately before the third season, explaining the within-season environmental change. Following the third season, (*o*) introduces the individualism-collectivism questionnaire, one question from which is shown in (*p*). These instructions were translated into Cantonese for the CM and HK groups (screenshots available upon request from the corresponding author).

Figure S6 (following pages). Screenshots of the task.

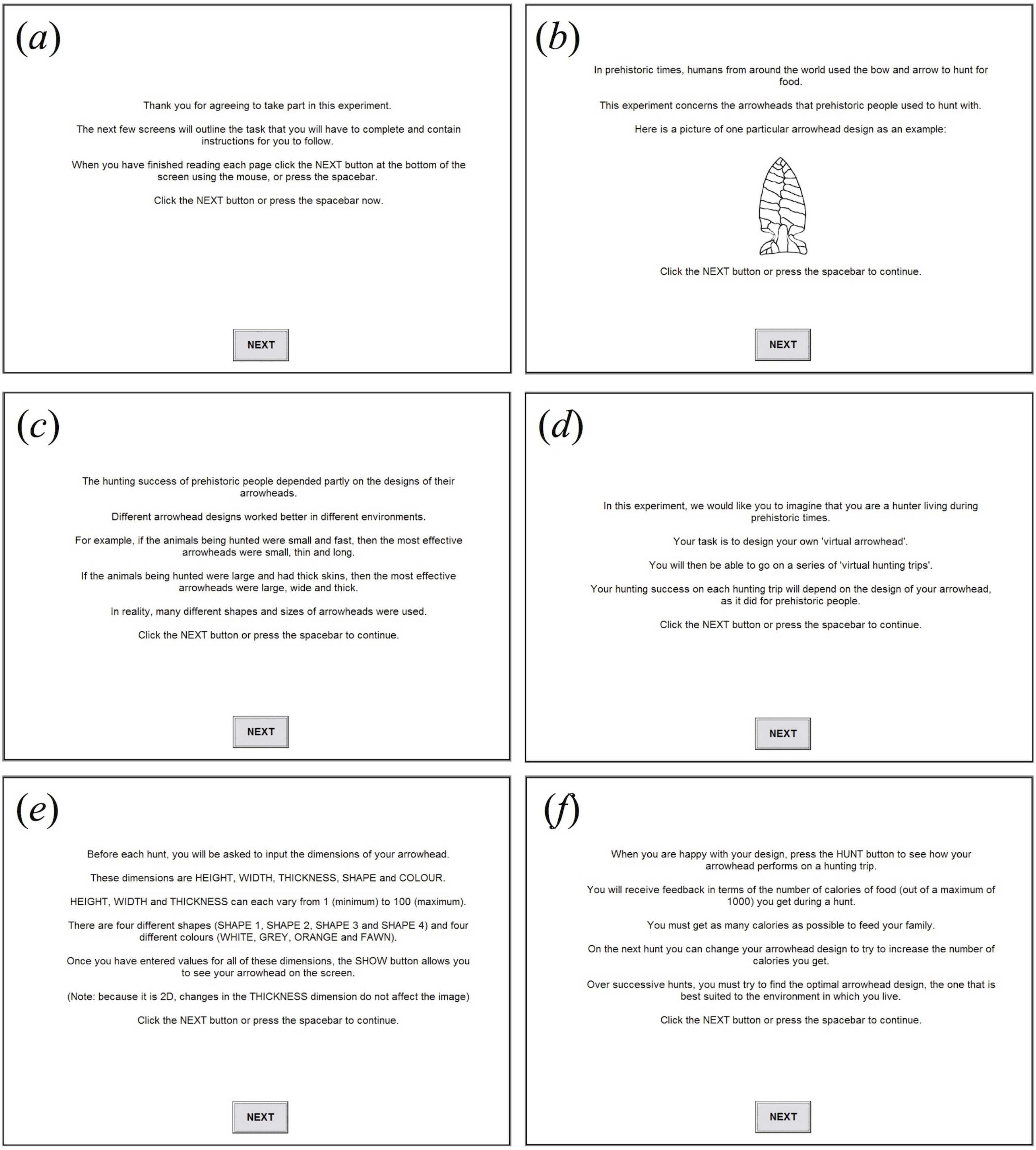
(a)
Thank you for agreeing to take part in this experiment.
The next few screens will outline the task that you will have to complete and contain instructions for you to follow.
When you have finished reading each page click the NEXT button at the bottom of the screen using the mouse, or press the spacebar.
Click the NEXT button or press the spacebar now.
NEXT
(b)
In prehistoric times, humans from around the world used the bow and arrow to hunt for food.
This experiment concerns the arrowheads that prehistoric people used to hunt with.
Here is a picture of one particular arrowhead design as an example:
Click the NEXT button or press the spacebar to continue.
NEXT
(c)
The hunting success of prehistoric people depended partly on the designs of their arrowheads.
Different arrowhead designs worked better in different environments.
For example, if the animals being hunted were small and fast, then the most effective arrowheads were small, thin and long.
If the animals being hunted were large and had thick skins, then the most effective arrowheads were large, wide and thick.
In reality, many different shapes and sizes of arrowheads were used.
Click the NEXT button or press the spacebar to continue.
NEXT
(d)
In this experiment, we would like you to imagine that you are a hunter living during prehistoric times.
Your task is to design your own 'virtual arrowhead'.
You will then be able to go on a series of 'virtual hunting trips'.
Your hunting success on each hunting trip will depend on the design of your arrowhead, as it did for prehistoric people.
Click the NEXT button or press the spacebar to continue.
NEXT
(e)
Before each hunt, you will be asked to input the dimensions of your arrowhead.
These dimensions are HEIGHT, WIDTH, THICKNESS, SHAPE and COLOUR.
HEIGHT, WIDTH and THICKNESS can each vary from 1 (minimum) to 100 (maximum).
There are four different shapes (SHAPE 1, SHAPE 2, SHAPE 3 and SHAPE 4) and four different colours (WHITE, GREY, ORANGE and FAWN).
Once you have entered values for all of these dimensions, the SHOW button allows you to see your arrowhead on the screen.
(Note: because it is 2D, changes in the THICKNESS dimension do not affect the image)
Click the NEXT button or press the spacebar to continue.
NEXT
(f)
When you are happy with your design, press the HUNT button to see how your arrowhead performs on a hunting trip.
You will receive feedback in terms of the number of calories of food (out of a maximum of 1000) you get during a hunt.
You must get as many calories as possible to feed your family.
On the next hunt you can change your arrowhead design to try to increase the number of calories you get.
Over successive hunts, you must try to find the optimal arrowhead design, the one that is best suited to the environment in which you live.
Click the NEXT button or press the spacebar to continue.
NEXT

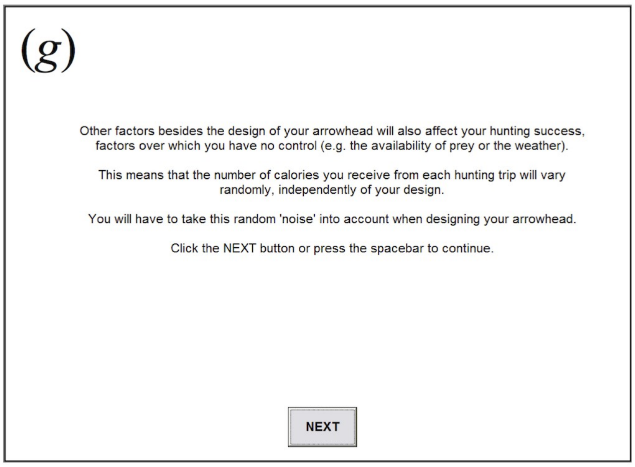
(g)
Other factors besides the design of your arrowhead will also affect your hunting success, factors over which you have no control (e.g. the availability of prey or the weather).
This means that the number of calories you receive from each hunting trip will vary randomly, independently of your design.
You will have to take this random 'noise' into account when designing your arrowhead.
Click the NEXT button or press the spacebar to continue.
NEXT

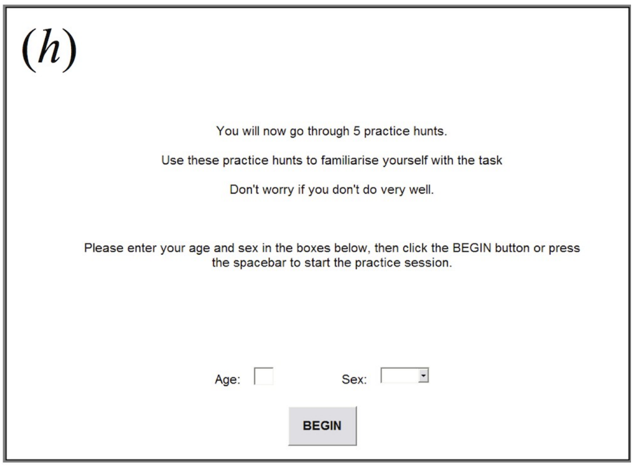
(h)
You will now go through 5 practice hunts.
Use these practice hunts to familiarise yourself with the task
Don't worry if you don't do very well.
Please enter your age and sex in the boxes below, then click the BEGIN button or press the spacebar to start the practice session.
Age:
Sex:
BEGIN

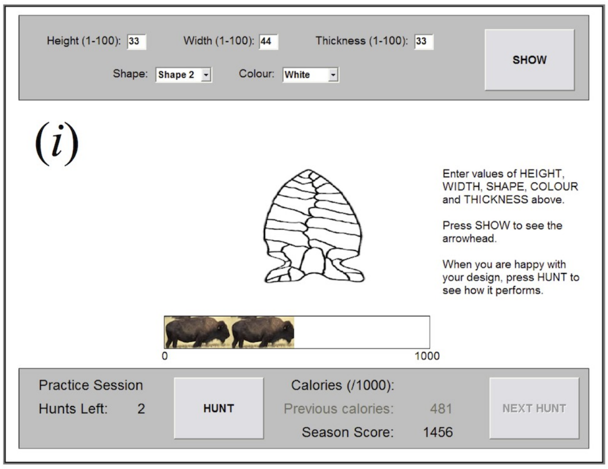
Height (1-100): 33
Width (1-100): 44
Thickness (1-100): 33
Shape: Shape 2
Colour: White
SHOW
(i)
Enter values of HEIGHT, WIDTH, SHAPE, COLOUR and THICKNESS above.
Press SHOW to see the arrowhead.
When you are happy with your design, press HUNT to see how it performs.
0
1000
Practice Session
Hunts Left: 2
HUNT
Calories (/1000):
Previous calories: 481
Season Score: 1456
NEXT HUNT

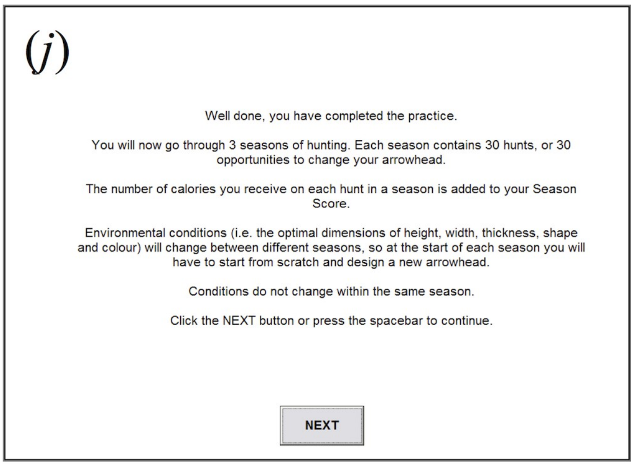
(j)
Well done, you have completed the practice.
You will now go through 3 seasons of hunting. Each season contains 30 hunts, or 30 opportunities to change your arrowhead.
The number of calories you receive on each hunt in a season is added to your Season Score.
Environmental conditions (i.e. the optimal dimensions of height, width, thickness, shape and colour) will change between different seasons, so at the start of each season you will have to start from scratch and design a new arrowhead.
Conditions do not change within the same season.
Click the NEXT button or press the spacebar to continue.
NEXT

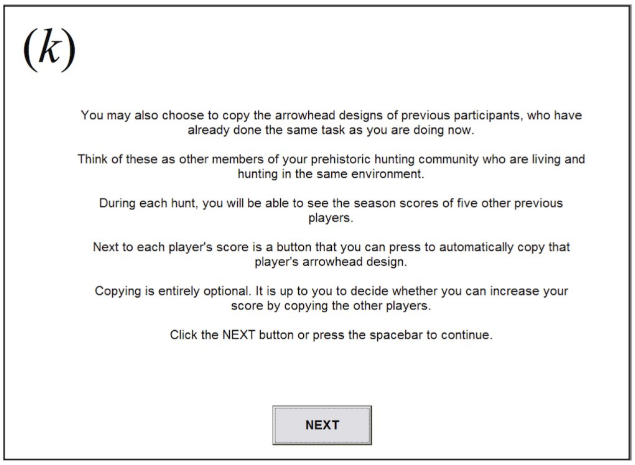
(k)
You may also choose to copy the arrowhead designs of previous participants, who have already done the same task as you are doing now.
Think of these as other members of your prehistoric hunting community who are living and hunting in the same environment.
During each hunt, you will be able to see the season scores of five other previous players.
Next to each player's score is a button that you can press to automatically copy that player's arrowhead design.
Copying is entirely optional. It is up to you to decide whether you can increase your score by copying the other players.
Click the NEXT button or press the spacebar to continue.
NEXT

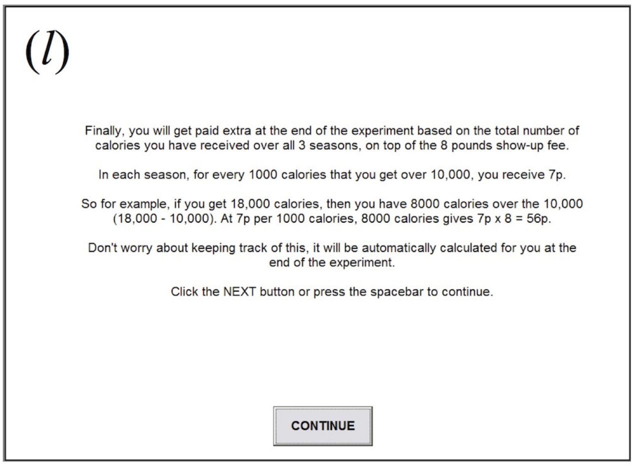
(l)
Finally, you will get paid extra at the end of the experiment based on the total number of calories you have received over all 3 seasons, on top of the 8 pounds show-up fee.
In each season, for every 1000 calories that you get over 10,000, you receive 7p.
So for example, if you get 18,000 calories, then you have 8000 calories over the 10,000 (18,000 - 10,000). At 7p per 1000 calories, 8000 calories gives 7p x 8 = 56p.
Don't worry about keeping track of this, it will be automatically calculated for you at the end of the experiment.
Click the NEXT button or press the spacebar to continue.
CONTINUE

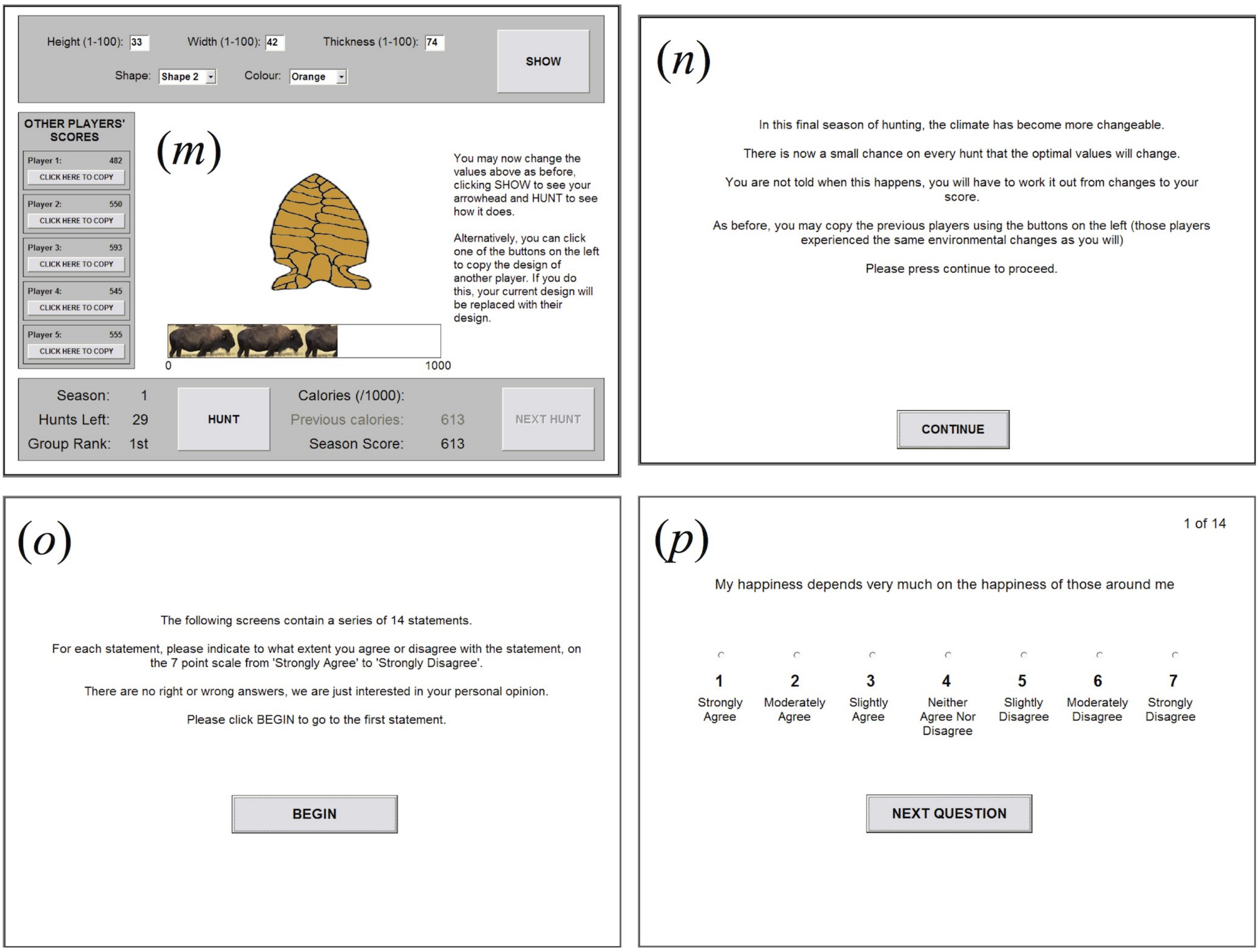
Height (1-100): 33
Width (1-100): 42
Thickness (1-100): 74
SHOW
Shape: Shape 2
Colour: Orange
OTHER PLAYERS' SCORES
Player 1: 482
CLICK HERE TO COPY
Player 2: 550
CLICK HERE TO COPY
Player 3: 593
CLICK HERE TO COPY
Player 4: 545
CLICK HERE TO COPY
Player 5: 555
CLICK HERE TO COPY
(m)
You may now change the values above as before, clicking SHOW to see your arrowhead and HUNT to see how it does.
Alternatively, you can click one of the buttons on the left to copy the design of another player. If you do this, your current design will be replaced with their design.
0
1000
Season: 1
Hunts Left: 29
Group Rank: 1st
HUNT
Calories (/1000):
Previous calories: 613
Season Score: 613
NEXT HUNT
(n)
In this final season of hunting, the climate has become more changeable.
There is now a small chance on every hunt that the optimal values will change.
You are not told when this happens, you will have to work it out from changes to your score.
As before, you may copy the previous players using the buttons on the left (those players experienced the same environmental changes as you will)
Please press continue to proceed.
CONTINUE
(o)
The following screens contain a series of 14 statements.
For each statement, please indicate to what extent you agree or disagree with the statement, on the 7 point scale from 'Strongly Agree' to 'Strongly Disagree'.
There are no right or wrong answers, we are just interested in your personal opinion.
Please click BEGIN to go to the first statement.
BEGIN
(p)
1 of 14
My happiness depends very much on the happiness of those around me
1 Strongly Agree
2 Moderately Agree
3 Slightly Agree
4 Neither Agree Nor Disagree
5 Slightly Disagree
6 Moderately Disagree
7 Strongly Disagree
NEXT QUESTION

References

1. Mesoudi, A. 2011 An experimental comparison of human social learning strategies: payoff-biased social learning is adaptive but underused. *Evol. Hum. Behav.* **32**, 334–342.
2. Paternoster, R., Brame, R., Mazerolle, P. & Piquero, A. 1998 Using the correct statistical test for the equality of regression coefficients. *Criminology* **36**, 859–866.
3. Sivadas, E., Bruvold, N. T. & Nelson, M. R. 2008 A reduced version of the horizontal and vertical individualism and collectivism scale: A four-country assessment. *J. Bus. Res.* **61**, 201–210.
